# Initial identification of genomic islands in *Taylorella equigenitalis* and *Taylorella asinigenitalis* and their distribution in isolates from around the world

**DOI:** 10.1186/s12866-026-05204-3

**Published:** 2026-06-05

**Authors:** Jessica Hicks, Ganwu Li, Sandrine Petry, Fabien Duquesne, Jun-Gu Choi, Gudrun Overesch, Kristina Lantz, Suelee Robbe-Austerman, Xiaoqiu Huang

**Affiliations:** 1 National Veterinary Services Laboratories (NVSL), USDA, 1920 Dayton Ave, Ames, IA 50010 United States of America; 2 Veterinary Diagnostic Laboratory, Iowa State University, 1937 Christensen Dr, Ames, IA 50011 United States of America; 3 Pathophysiology and Epidemiology of Equine Diseases Unit, Laboratory for Animal Health, ANSES, Dozulé, Goustranville, 14430, 610101 France; 4 Foreign Animal Disease Division, Animal and Plant Quarantine Agency, 177, Hyeoksin 8-ro, Gimcheon, 39660 Gyeongsangbuk-do Republic of Korea; 5 Institute of Veterinary Bacteriology, University of Bern, 122 Laenggassstrasse, Bern, 3012 Switzerland; 6 Department of Computer Science, Iowa State University, Ames, IA 50011 United States of America

**Keywords:** Taylorella equigenitalis, Taylorella asinigenitalis, CEM, Genomic islands, Virulence factors, Secretion systems

## Abstract

*Taylorella equigenitalis*, the causative agent of contagious equine metritis (CEM), is endemic in several countries throughout the world and impacts the global movement of horses. It presents with symptoms ranging from non-clinical to endometritis, vaginitis, and acute infertility in females, while males remain asymptomatic. Studies of the disease have focused primarily on understanding pathology, transmission dynamics, diagnostics, and phenotypic and genotypic characteristics. The goal of this study was to establish a more comprehensive global phylogenetic structure and generate reference-quality genomes for each major clade of the tree where such data were absent. Seven of these genomes along with 44 other publicly available genome assemblies, including *Taylorella asinigenitalis*, were then used to predict genomic islands. Exploration of the NCBI annotations in the genomic islands indicate links to virulence and ecological fitness. An additional 97 *T. equigenitalis* and 48 *T. asinigenitalis* short read sequenced isolates from this study and 215 publicly available genomes were used to examine distribution of each genomic island across the phylogenetic tree for evidence of horizontal movement. The stability of these genomic islands in their natural environment was evaluated utilizing 141 isolates recovered from a recent CEM outbreak in the United States. The results obtained in this study demonstrate mobile genomic islands in *T. equigenitalis* genomes, sites of concern for their contribution to the potential virulence of the organism.

**Supplementary Information:**

The online version contains supplementary material available at 10.1186/s12866-026-05204-3.

## Introduction

 Contagious equine metritis (CEM), a disease caused by the bacterium *Taylorella equigenitalis* (TE), was first reported in 1977 and remains an international trade concern in the horse industry [1]. In females, the disease manifests as copious vaginal discharge, vaginitis, and endometritis, sometimes resulting in acute infertility; but symptoms present with varying degrees of clinical illness from severe to subclinical. Males are asymptomatic carriers of the bacteria making them a significant concern in disease transmission [2–5]. Global movement of horses and the often-low numbers of colonized bacteria on males allows many opportunities for the spread of *T. equigenitalis*. This is World Organization Animal Health (WOAH) listed disease, and detections are therefore reportable.

Several studies have sought to characterize TE using various approaches, ranging from in vitro cellular infiltration to genotyping techniques such as pulse-field gel electrophoresis (PFGE) and multi-locus sequence typing (MLST) [6–8]. These methods have been instrumental in grouping strains and improving epidemiological tracing but fall short in molecular characterization of individual strains and identifying their potential virulence factors. Although, basic molecular characterization, such as 37.5% average whole genome GC content and a genome length of approximately 1.6–1.8 megabase pairs, can be derived from data made available through these studies. A recent phylogenetic analysis of 28 TE genomes revealed that, on average, 6.6% of each genome potentially contained recombination events [9]. Genomic comparisons between TE isolates have uncovered intraspecies variation, specifically within discrete regions of diversity and potential mobile elements that have remained uncharacterized [10–12]. Some genes within these variable regions are associated with virulence, including hemagglutinin proteins, which function as mammalian cell adhesins in many bacteria; type IV secretion systems (T4SSs), which mediate effector protein delivery across the cell membrane and facilitate the uptake of environmental proteins or DNA; and CRISPR-Cas systems [10–14]. However, it remains unclear whether these variable regions arise from mobile elements or other sources of genetic variation. Moreover, these genomic comparisons have been limited to a small number of isolates, potentially underrepresenting the species’ genomic diversity. A comprehensive study analyzing isolates from diverse geographic regions is yet to be conducted. Expanding our understanding of TE genomics and virulence factors and their mobility between strains could lead to advancements in diagnostic assays and a deeper comprehension of the organism’s disease cycle.

*Taylorella asinigenitalis* (TA), the only other species in the genus *Taylorella*, is generally considered non-pathogenic [15–17]. However, some strains have shown the potential to cause mild clinical illness and infection of mares [18, 19]. Relatively little has been done to establish genomic diversity or understand phylogenetic structure, and relatively little public data exists for this organism. An initial genome comparison of a single strain of TA (MCE3) with a single strain of TE (MCE9) indicated a large overlap in genes, with 1322 of the 1534 total genes annotated in TA being shared with TE; but it is unknown if this overlap includes genetic mechanisms that contribute to virulence. Also, a large inversion in the TA genome relative to TE was noted and a similar genome size with this assembly being 1,638,559 bp [11]. However, the average whole genome GC content in this assembly varies from that of TE with 38.3%.

Genomic islands (GEIs) are broadly defined as large, mobile gene clusters within a genome and can include many types of mobile elements, e.g., prophages, integrative conjugative elements, integrons, conjugative transposons, among others [20, 21]. GEIs can considerably enhance the genetic potential of an organism and have several functional classifications such as pathogenicity islands (PAIs), ecological islands, and other types related to metabolism, symbiosis, and antimicrobial resistance [20, 22]. GEIs are considered to contribute to genomic plasticity with their ability to easily move sets of genes in and out of the genome as needed in order to adapt to environmental challenges [20]. Often, GEIs occur near tRNAs, differ in GC content from the rest of the genome, and contain mobility genes such as integrases or translocases [20]. Prediction of GEIs is accomplished through a variety of different algorithms used to detect regions that meet these characteristics: comparison to known GEIs, presence of known mobility genes in combination with nucleotide biases, and hidden Markov model approaches [23, 24].

The first goal of this study was to establish a more comprehensive global phylogenetic structure. Seven new clades consisting of the sequences from this study were added to the TE tree and complete, reference-quality genomes for these clades were created. Secondly, the reference sequences along with 45 other publicly available genome assemblies, including TA, were analyzed to predict GEIs and examine gene content. While National Center for Biotechnology Information (NCBI) annotations of the predicted GEIs reveal potential links to virulence and ecological fitness, the abundance of hypothetical proteins underscores the potential for additional, unidentified functions. To investigate conservation and distribution of the GEIs across the phylogenetic tree, an additional 97 TE and 48 TA short-read isolates from this study alongside 215 publicly available genomes were examined. Thirdly, to explore this dynamic in natural settings, 141 TE isolates recovered from a recent CEM outbreak in the United States were evaluated for presence and absence of the GEIs, as well as the combinations of GEIs found in these isolates. The results obtained in this study demonstrate the mobility of the predicted GEIs in TE genomes and their potential contribution to virulence of the organism.

## Materials and methods

### Isolate selection

To better characterize the global phylogeny, collaborators contributed isolates collected from Belgium, France, the Netherlands, South Africa, South Korea, Switzerland, the United Kingdom, and the USA. Each country selected isolates that represented the most diversity for their isolate collection, either by MLST or by geographical region, or sent their entire collection to the National Veterinary Services Laboratories (NVSL) in Ames, Iowa USA for subculture and sequencing. In addition to a previously sequenced culture collection of TE isolates from domestic and imported detections in the USA, additional isolates more recently recovered from imported horses (*n* = 97 TE) and from a recent outbreak (*n* = 141 TE) within domestic ponies and horses were selected. All TA isolates from the NVSL reference culture collection and a single isolate submission from France were also included in this study (*n* = 48 TA).

### Culture, DNA extraction, and sequencing

Isolates were obtained from bacteriological culture at the NVSL or shipped as frozen isolates. Isolates were subcultured on chocolate Eugon agar with 5% horse blood from frozen culture and incubated for 48–72 h at 37 °C in 5% CO_2_. Colonies were selected and extracted using either the Epicenter Masterpure kit (Epicentre, Madison, WI, USA) or a Promega Maxwell with Maxwell Whole Blood DNA Kit (Promega Corporation, Madison, WI, USA) at the suggestion of the manufacturer and according to their instructions. DNA library preparation was performed with a Nextera XT kit v2 with set B (CD indices) (Illumina, San Diego, CA, USA), and sequencing was performed on an Illumina MiSeq with a 2 × 250 bp chip or an Illumina NextSeq with a 2 × 150 bp or 2 × 250 bp chip. A minimum depth of 70X coverage was targeted with only 4 TE isolates in this study having less (33X-60X). Coverage statistics for these isolates were carefully examined to ensure patterns were consistent with deeper coverage isolates. Isolates selected for long-read sequencing were propagated in the same manner, extracted using PureGene (Qiagen, Hilden, Germany), and sequenced on a PacBio Sequel (Pacific Biosciences, Menlo Park, CA, USA).

### Evaluation, assembly, and phylogenetic analysis

Short-read sequences were screened for purity using Kraken and a custom database that includes the NCBI RefSeq database as well as potential host references [25]. Short-read sequences were then *de novo* assembled with Spades [26]. Additional TE and TA sequences in NCBI were downloaded and with the Spades assemblies of both *Taylorella* species, a reference independent maximum likelihood phylogenetic tree was generated using kSNP with a 0.8 majority setting (to decrease influence of individual variations, mobile elements, and potential sources of contamination on the resultant tree), and allowing KChooser to optimize the k parameter, and maximum likelihood tree was used [27]. This analysis was used to select isolates for long-read sequencing to develop references for each major branch of the tree. Once this sequencing was completed, a TE-only tree was also constructed using kSNP.

Long-read sequences were assembled with Canu (version 2.0) and Unicycler (version 0.4.9) [28, 29]. Assembler outputs of each isolate were compared with each other using Mauve to examine identify any discrepancies between assembler outputs and subsequently to review circularization, which were resolved with short-read support through Pilon (version 1.22 with default settings) polishing and manual review in Integrated Genome Viewer (IGV) [30–33]. To circularize chromosomes, the ends of contigs were manually aligned to determine overlap and were trimmed accordingly. Once initial polishing rounds were completed, genomes were rotated from a random midpoint and short-read polishing was again carried out. All assemblies were oriented to the same start point to match previously published sequences, which is *dnaA*. To further improve the assemblies, short-read sequences were aligned to the long-read assembly of the same isolate using the vSNP (Step 1); and Pilon with default settings was used to generate consensus base calls [31, 34]. Iterations of alignment and consensus calling were repeated until no SNP changes were observed between iterations. Final assemblies were uploaded to NCBI along with the raw data, and annotation with PGAP was completed [35]. Sequencing statistics for all samples (long and short reads) are in Supplemental Tables 1 and 2.

### Core genome determination and lineage analysis

Fasta files of *de novo*, short-read assemblies of TE and complete assemblies of TE were annotated with PROKKA (version 1.14.6, https://github.com/tseemann/prokka) (regardless if NCBI annotations existed) to ensure consistency in the annotation since PGAP annotations from NCBI were not available for all isolates. Panaroo (version 1.5.2), with the clean-mode set to strict to remove potential sources of error, was used to determine core and pangenomes between all isolates in the study, and a core genome alignment with MAFFT (version 7.505) was output. IQTree (version 2.4.0, TVM + F+ASC+R2 selected based on Bayesian Information Criterion) was used with model selection to generate a phylogenetic tree based on the core genome alignment [36–39]. This tree was then used to perform lineage analysis with Fast BAPS (version 1.0.8) using the optimise symmetric setting [40].

### NCBI data

All data used in this study are listed in Supplementary Tables 1 and 2 and has been made public through NCBI repositories. For the purposes of developing a more complete phylogeny and predicting GEIs, a dataset defined here as the ‘global dataset’ was used. This dataset was comprised of the NCBI assemblies (7 TE genomes closed for this study and submitted to NCBI for annotation as well as 44 additional TE isolates and 1 TA isolate from the NCBI Assembly database) and NCBI Sequence Read Archive short-read data (215 Illumina sequenced isolates and an additional 97 TE and 48 TA isolates sequenced for this study) [35, 41]. To examine the behavior of the GEIs under normal environmental conditions, 141 isolates from a recent US outbreak were used, a dataset defined here as the ‘outbreak dataset’.

### Predicting genomic islands

IslandCompare was used to search the assemblies in the global dataset for GEIs using the GenBank annotation files from NCBI [42]. IslandViewer4 was then used to generate genome plots of the assemblies closed for this study with GEI locations, and through this process an additional potential GEI was identified (Supplemental Fig. 1). Classification and further refinement of the GEIs was performed with ICEberg 3.0s ICEfinder, a tool specifically aimed at classifying integrative and conjugative elements (ICE), integrative and mobilizable elements (IME), and cis-mobilizable elements (CIME) [43]. Each TE GEI in each isolate was compared with others of the same type identified in other isolates. Margins of the GEIs were evaluated to ensure consistency across isolates and were manually adjusted as warranted by homology to genes included in the genomic island in other isolates. GEIs were then extracted and aligned using MAFFT in Geneious Prime version 2022.1.1 to generate identical sites per genomic island [37, 44]. Final versions of each assembled GEI were aligned in pyGenomeViz for visualization (https://github.com/moshi4/pyGenomeViz).

A representative sequence of each GEI was compiled into a single fasta-formatted file for an alignment reference with the short-read sequences in the global dataset. Each short-read sequenced isolate was aligned to the compiled reference fasta with vSNP Step1, which uses BWA-MEM for alignment, FreeBayes for SNP calling, and outputs alignment metrics [34, 45]. Additional alignment statistics, such as percent coverage, mean base quality, and mean map quality, were obtained using Samtools coverage [46]. Compiled statistics for the GEIs were evaluated for percent coverage and depth with at least 80% identity; and for four of the six TE GEIs (Groups 1–3, and 5), isolates primarily clustered into 2 distinct groups by percent coverage alone. For the 4th and 6th GEIs, there was no clear division in coverage to establish presence or absence; and visual inspection of alignments between isolates predicted to carry a GEI and those without the prediction show variation in a region primarily containing rearrangement hot spot genes. When isolates were evaluated for coverage specifically in this region, two coverage groups were formed. To ensure that the coverage groups correlated with presence/absence of each GEI, short-read sequences of the assemblies (where available) were aligned to the compiled GEI reference. These were then evaluated to establish appropriate coverage cutoffs for inclusion/exclusion. The presence/absence determination based on this short-read data was compared with predictions on the assemblies to ensure consistency in predictions.

### Stability of genomic islands

The outbreak dataset was used to examine the presence of GEIs in this clonal dataset that came from natural infection having been passed to several animals over a period of years in the natural environment. Caution was used to avoid serial passaging of cultures prior to DNA extraction, which attempted to avoid laboratory adaptation and the possible loss of GEIs under laboratory conditions. Sequences were aligned to the GEI reference in the fasta format used previously, and isolates were characterized for the presence and absence of each genomic island as described above. All outbreak isolates belong to MLST 50, and to further illustrate the genetic relatedness of isolates, vSNP was used with reference NZ_CP0076337 to generate a SNP-based phylogeny [34]. Initially, the tree was built using all SNP data including GEIs; however, a second tree was generated with SNP data throughout the GEIs filtered from the dataset to visualize genetic relatedness without the influence of the mobile elements.

## Results

### Phylogenetics

**Fig. 1:**
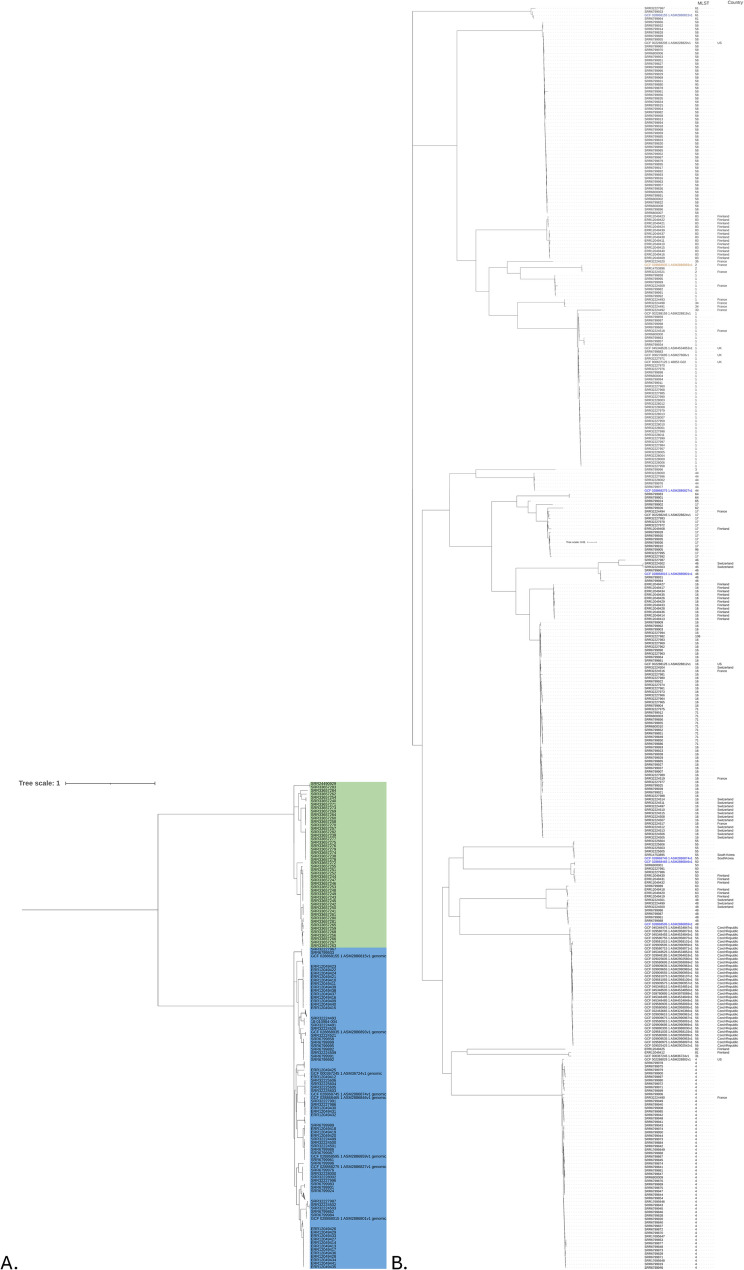
kSNP majority phylogenetic trees. **A** TA and TE isolates from the global dataset. The top clade is comprised of TA isolates while TE is in the bottom clade. NCBI Assemblies are highlighted in blue. **B** TE phylogenetic tree from kSNP with 0.8 majority used with isolates from the global dataset. Isolates in blue are NCBI Assemblies, green branches indicate clades with a closed reference from this study, the orange branch indicates the placement of the re-sequenced MCE9 isolate from this study

**Fig. 2:**
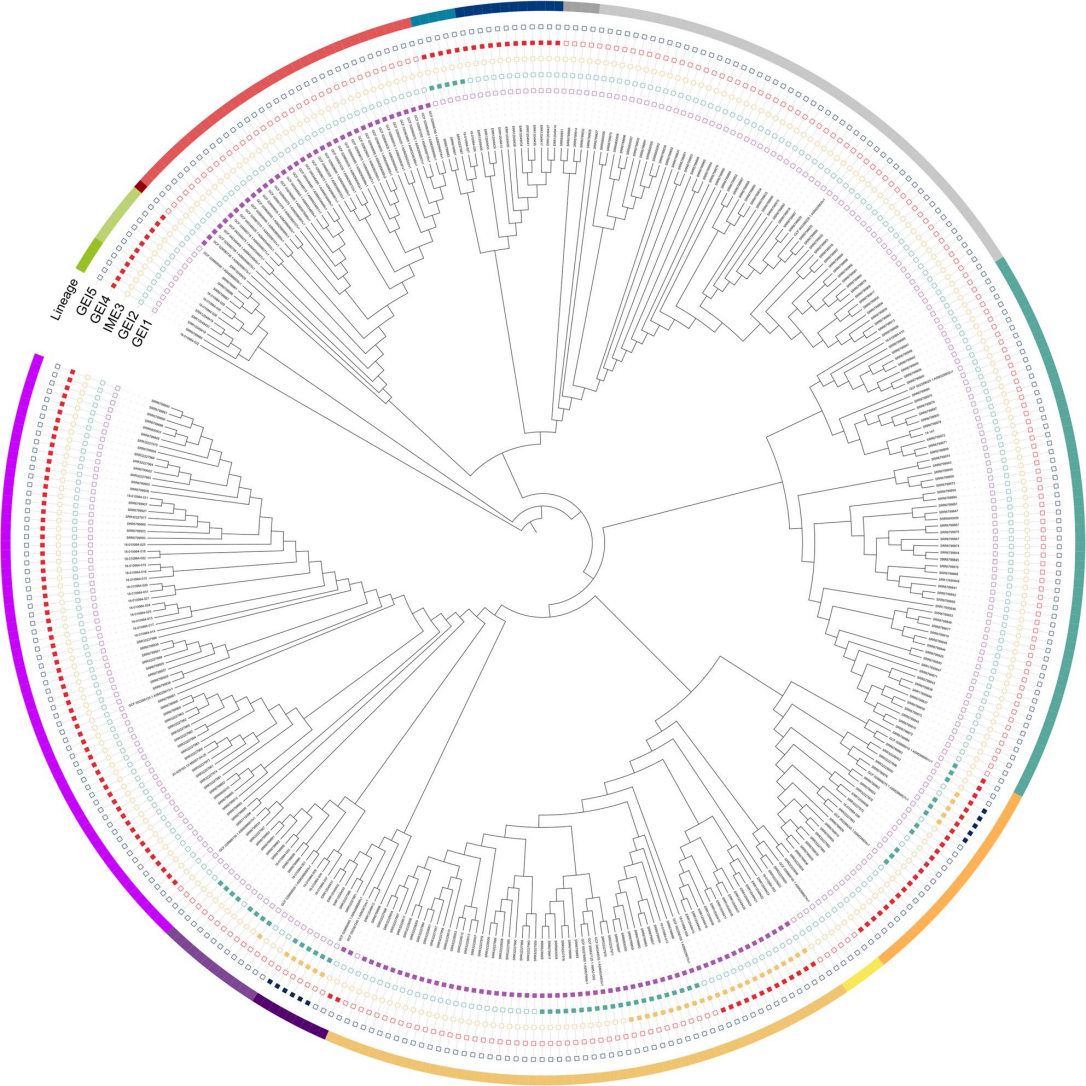
Core genome phylogenetic tree. This tree, generated by IQTree, shows the 2-level lineage analysis and presence of GEIs 1–5. Each level 1 lineage is denoted by color category with shading representing level 2 sublineages

The kSNP tree of both *Taylorella* species shows distinct separation between TE (global dataset only) and TA (Fig. 1A); and, within the TE tree (global dataset only), the isolates are sorted into highly divergent clades (Fig. 1B). Overall, the trees show significant genetic diversity while clustering previous outbreak isolates as well as some isolates with common geographic origins. The long, internal branches are consistent with results of the recent Czech study which places the most recent common ancestor of TE around 640 A.D. (80–900 A.D.) [9]. The 7 closed genomes from this study provide the first closed reference for 6 clades as well as re-sequencing of MCE9 in the kSNP tree in Fig. 1. The clustering of the complete genomes in the core genome tree in Fig. 2 does vary, expectedly, from the clustering in Fig. 1.

### Core genome and lineage analysis

The TE core genome, which is defined by genes occurring in ≥ 99% of genomes, contained 1404 genes, which comprised 70.5% (1404/1992) of the total genes identified in this species. The average GC content of the core genome identified in this analysis was 38.0%. Alignment of these genes and subsequent lineage analysis yielded 7 main lineages that further divided into 15 sub-lineages as seen in Fig. 2. These occurred along major branches of the core genome phylogenetic tree. Six of the seven main lineages all contain complete genome assemblies, while only 10 of the 15 sublineages contain complete genome assemblies. It should be noted that 4 of the 5 sublineages not containing a complete assembly are comprised of 5 or fewer isolates. Placement of the GEIs in the core genome tree shows broad dispersion across the tree, even though these GEIs do not occur in the core genome since none of them are present is 99% of the isolates.

### Genomic islands

A total of 7 GEIs were identified with 6 being found in only TE genomes and 1 in the TA genome. IslandCompare identified 5 unique GEIs (named GEI1 to GEI5) across all 51 TE assemblies with each genome having 0–4 GEIs (Supplemental Table 3). IslandViewer4, based on 12 assemblies completed by the NVSL, predicted the same GEIs as IslandCompare plus a potential 6th TE GEI (named GEI6) (Supplemental Fig. 1). ICEberg’s ICEfinder refined the positions of 2 GEIs, GEI2 and GEI3, and provided classifications of both GEIs. Regardless of prediction tool, GEIs of the same group show a high degree of similarity across genomes as seen in Table 1. GC content of each predicted GEI is also listed in table for comparison against the average whole genome GC content of 37.5% and average core genome GC content of 38%. Annotations of each GEI are shown in Supplemental Table 1.

**Table 1:** General Features of the TE GEI Detected in this study and number of isolates per GEI: Table 1 shows the average GC content and length of each GEI as well as the number of assemblies with each GEI and the percent of identical sites across those GEIs when aligned with MAFFT. It also shows the number of short read sequenced (SRS) isolates with each GEI and the breakdown of coverage for considering the GEI present or absent in each GEI

Number of Isolates with GEI								
	Average GC	Average Length (bp)	Assemblies with GEI	Identical Sites in Assemblies	SRS Isolates with GEI	Coverage Range for Present	Coverage Range for Absent	TA Coverage Range
GEI1	32.0%	8,964	40	97.3%	57	97–100%	11–75%	8–23%
GEI2	32.7%	40,946	9	76.8%	47	83–100%	2–8%	2–42%
IME	30.7%	7,281	3	97.90%	34	83–100%	0–17%	0–41%
GEI4	37.4%	16,462	5	70.90%	131	99.9–100%*	3–38%*	32–53%
GEI5	34.5%	10,047	2	99.90%	11	99.8–100%	26–61%	24–49%
GEI6	37.7%	44,846	5**	60.50%	**	**	**	50–78%
*evaluated from position 1000 to 2700								

** Only the 12 assemblies from the NVSL were included in this prediction. Presence/Absence metrics could not be determined

#### Genomic island 1

GEI1 was the most prevalent GEI in the assemblies, occurring in 40 of the 51 TE assemblies evaluated. However, this may be due to sampling bias as 35 of the 40 assemblies containing this GEI occur in a single clade. All 40 assemblies represent only 4 clades labeled in the TE tree (Fig. 2). GEI1 remained highly conserved across the 40 isolates with only 6 unique sequences observed and 98.1% identical sites between them. The 6 sequences can only be differentiated by SNPs and small inserts and deletions, but annotations (Supplemental Table 1) remained the same between all the sequences. The initiation of this GEI was always located between 1.28 Mb and 1.35 Mb with a PD-(D/E)XK nuclease family protein at the 5’ end and N-acetylmuramoyl-L-alanine amidase at the 3’ end. The exact placement of this GEI in the genome was obviously influenced by the presence of other GEIs.

**Fig. 3:**
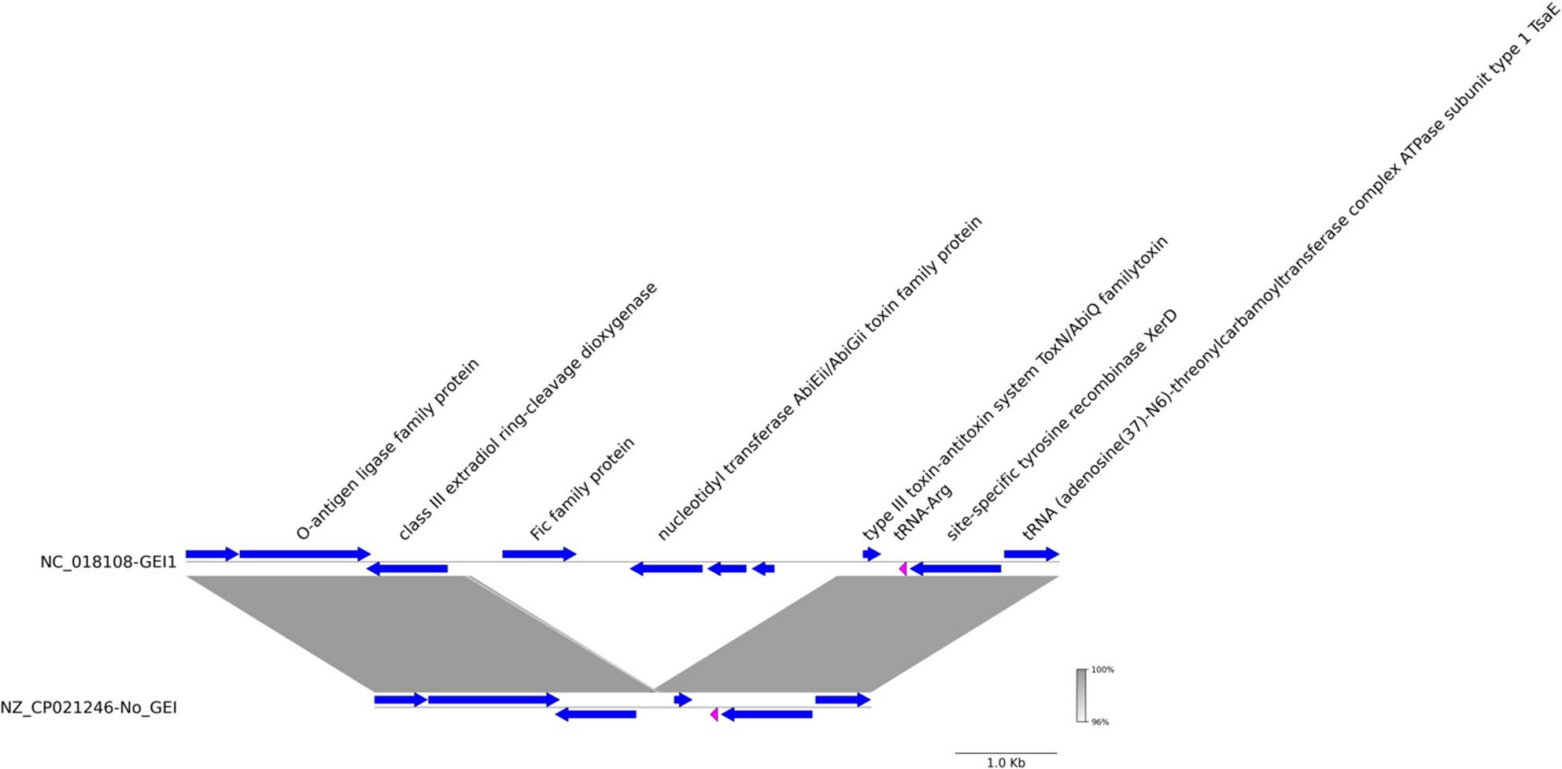
Alignment of GEI1. GEI1 as predicted by IslandCompare and IslandViewer4 in isolate NC_018108, while isolate NZ_CP021246 is representative of TE isolates without the GEI predicted. These 2 annotation patterns were the only patterns observed. Genes without annotation are hypothetical. Aligned segments represent 96% or greater BLAST matches

Among the TE short reads, coverage statistics for GEI1 fell into 2 groups: 97–100% and 53–75%, and only 1 isolate had less than 53% coverage. Fifty-seven isolates carrying the GEI1 were in the 97–100% group with an overall average depth of coverage of 94X, making this the second most common GEI identified in the short reads. However, the predicted GEI was rarely entirely absent in any isolates examined. As shown in Fig. 3, a truncated GEI1-like sequence was observed in 261 isolates, which comprised the group with 53–75% coverage. These isolates had an average depth of coverage of 82X. The deleted sequence in GEI1 spanned from a gene encoding the Fic family protein to the hypothetical protein located at the 3’ end of the *toxN* gene.

Conversely, alignment of the TA short reads to this same reference showed low coverage at 8–23% but greater than 90% identity in the coverd regions.

#### Genomic island 2

**Fig. 4:**
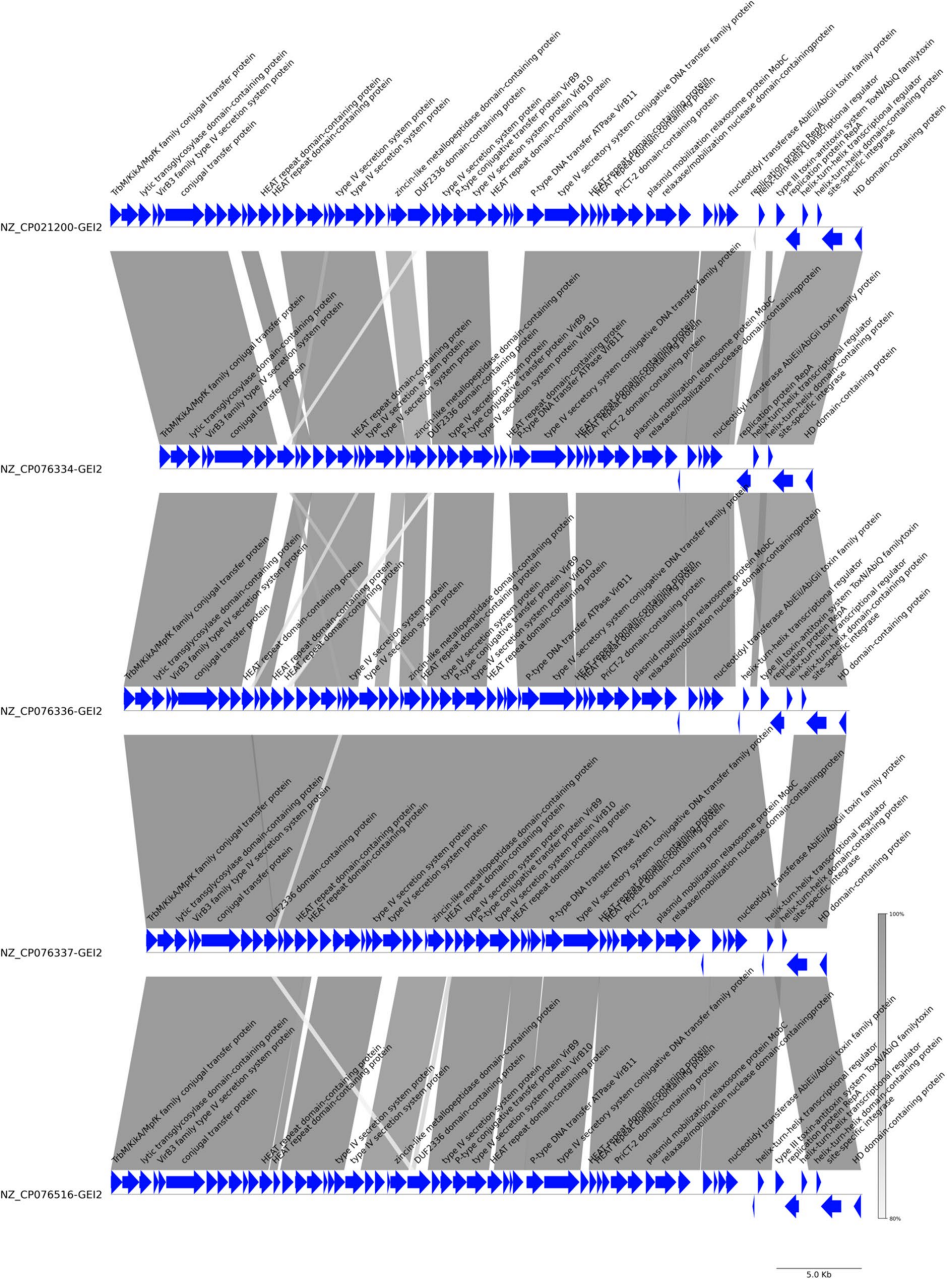
Alignment of GEI2. Among the assemblies with this genomic island, 5 variations in the annotations were found. The alignment of these sequences and annotations shows overall homology with discrete changes in the island. Genes without annotation are hypothetical. Aligned segments represent 80% or greater BLAST matches

This is the most widely dispersed GEI in the TE tree (Fig. 2), being found in isolates from 7 of the 13 major clades. GEI2 is the largest of all the GEIs identified in this study, classified as a T4SS-ICE by ICEberg. The 9 assemblies containing this GEI showed variability, with 5 different annotation patterns (Fig. 4). Notably, 3 isolates lacked a contiguous genomic region near the 3’ end containing the ToxN/AbiQ family toxin gene, the *repA* gene, and a helix-turn-helix transcriptional regulator. Despite this 2,556 bp gap, the GEI is composed of 76.8% identical sites across the 9 isolates, and it was consistently located within genomes with the 5’ end of GEI2 always located between 1.17 Mb and 1.21 Mb in the genome with a P-loop NTPase at the 5’ end and an intergenic region, sometimes containing a small hypothetical protein, followed by *dapA* at the 3’ end. Among the short reads, the coverage statistics formed 2 distinct groups: 83%-100% and less than 10%, with 47 isolates falling into the first group and 272 isolates in the second group.

Among the TA isolates, average coverage ranged from 2 to 42% in isolates with 37 of the 47 isolates having 5.3% or less coverage. All TA isolates had greater than 90% identity within the covered regions, regardless of percent coverage.

#### Genomic island 3

**Fig. 5:**
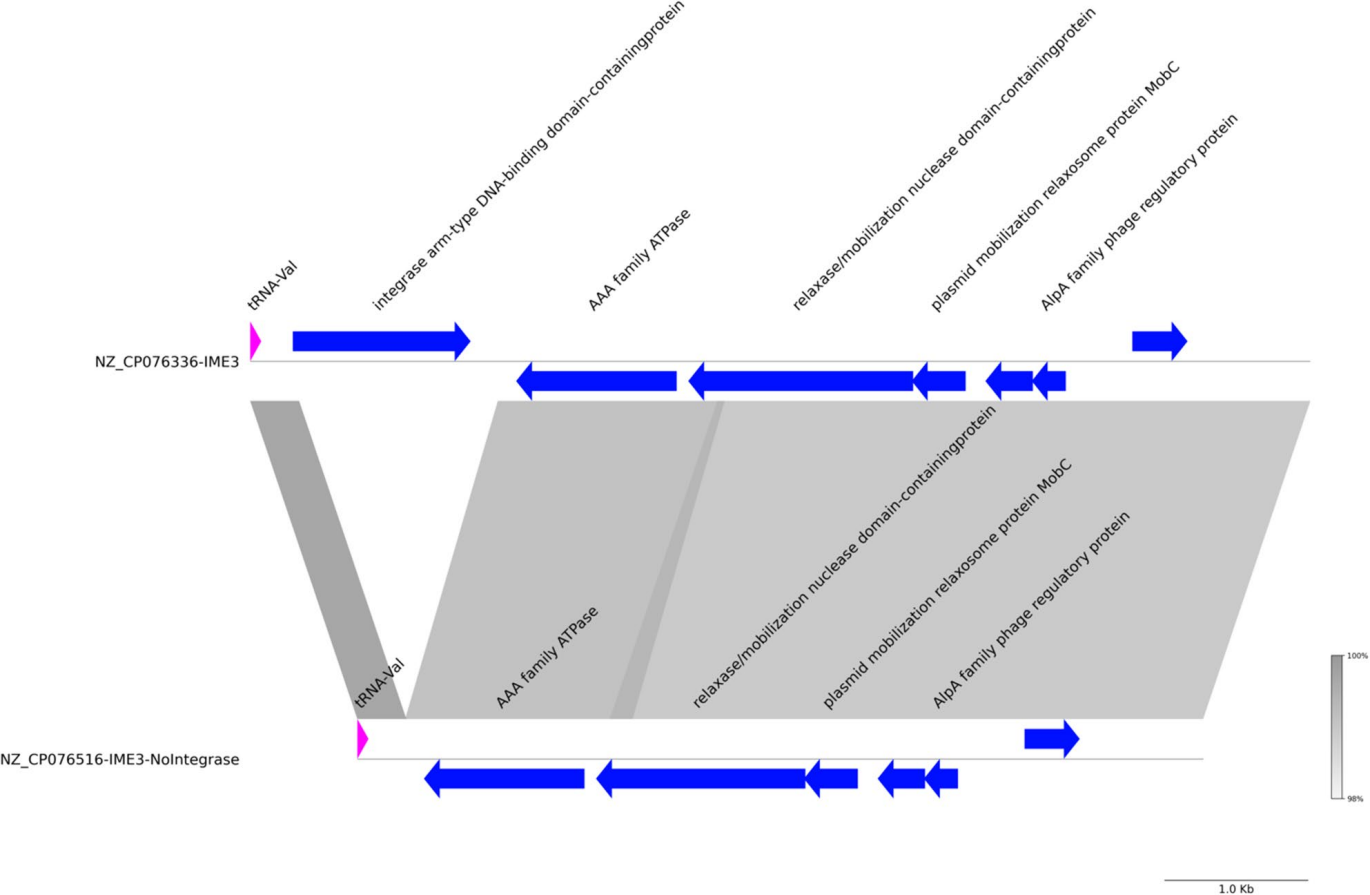
Alignment of sequences representing IME3, and the IME without integrase. NZ_CP076336 contained the full IME3, while NZ_CP076516 is missing the integrase within the IME. Genes without annotation are hypothetical. Aligned segments represent 98% or greater BLAST matches

GEI3 was predicted in 4 annotated genomes by IslandCompare. When these genomes were analyzed in ICEberg’s ICEFinder, a shifted, more refined region that included the site-specific integrase and AAA family ATPase immediately upstream of the IslandCompare prediction was classified as an integrated mobilizable element (IME). However, the integrase is not present in 1 of the assemblies, leaving only 3 assemblies with the IME identified (Fig. 5). As with the previous GEIs, it was consistently located in the same region of each genome beginning between 0.97–0.98 Mb in the genome and was flanked by *ung*, uracil-DNA glycosylase at the 5’ end and tetratricopeptide repeat protein at the 3’ end. Of the short reads, 34 had 98% or greater coverage to the IME. One additional short read isolate did not have the site-specific integrase (83% coverage). There was a significant drop in coverage in the remainder of the isolates - primarily ≤ 10%, with only 2 isolates showing slightly higher values at 14% and 17%.

Most TA isolates had 10% or less coverage of this GEI, but 4 isolates had 21%-25% coverage, and a single isolate had 41% coverage. While percent identity varied widely among isolates with the lowest coverage, the 5 isolates with higher coverage all had 89% or greater identity in the covered regions.

#### Genomic island 4

GEI4 had greater than 85% coverage in nearly all assemblies and short read sequences. The predominant region of variability was near the 5’ end in a set of genes annotated as hypothetical protein and Rearrangement Hot Spots, which appears to have separated assemblies with the predicted GEI from those without. Outside of this region, isolates contained genes encoding a transposase as well as TssI/VgrG, ATP binding protein, CstA (pyruvate/proton symporter), and TcuAB (tricarballylate utilization 4Fe-4 S protein and tricarballylate dehydrogenase). The *tcuA* gene contained a PAAR domain (proline-alanine-alanine-arginine), an essential counterpart to Tssi/VgrG to form the VgrG spike of the type VI secretion system (T6SS) [13]. A quick search of all the annotations (TE and TA) revealed the presence of this protein in all assemblies; however, this was not consistent in the short-read sequences as 4 TA isolates had no coverage of the entire region.

**Fig. 6:**
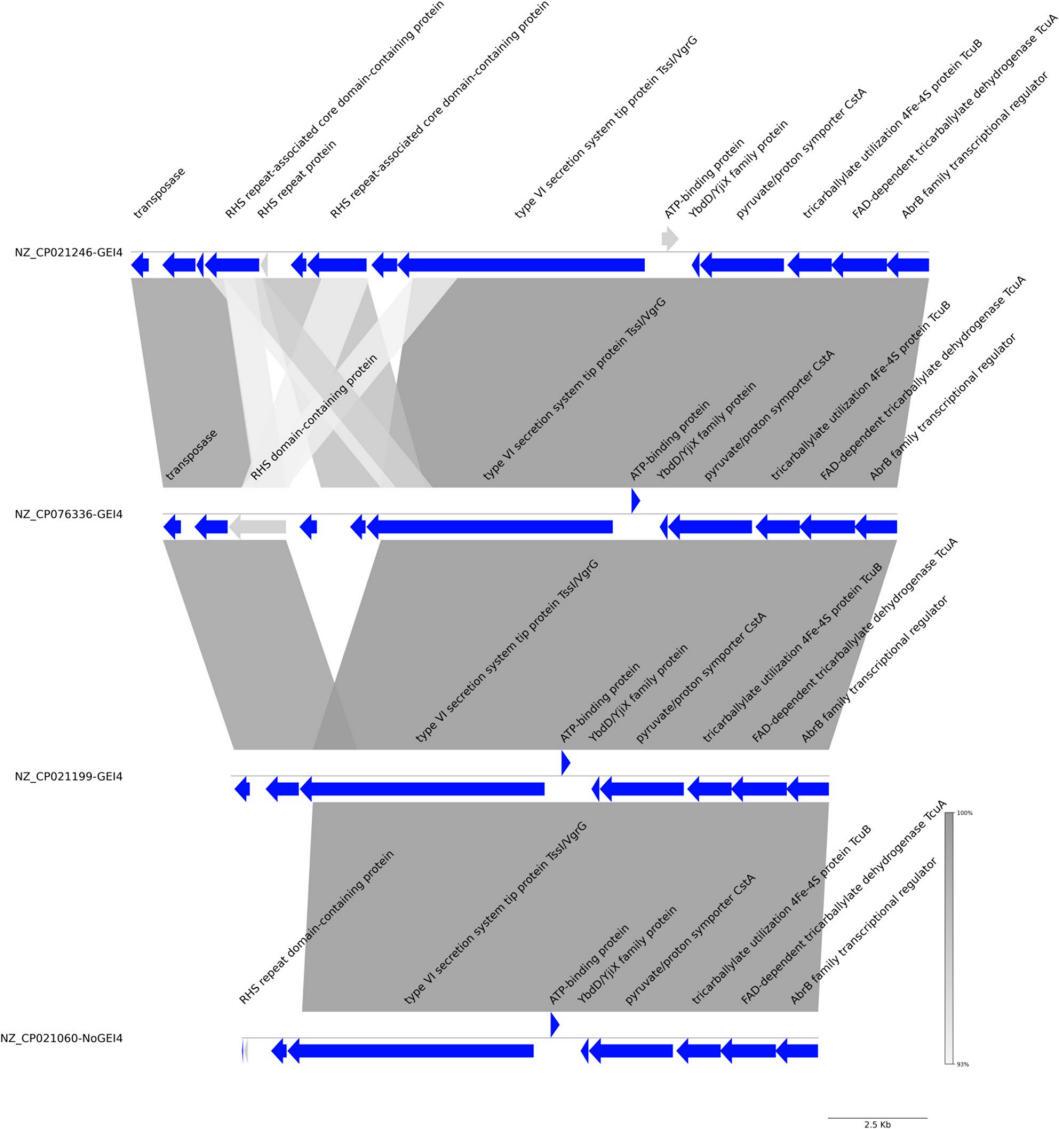
Alignment of GEI4 Sequences with a sequence found in the same region not predicted to be GEI4. Four annotation variations were observed for this GEI. NZ_CP021060 contains a sequence in the same region of the genome and with homology to a portion of GEI4 observed in other genomes. Genes without annotation are hypothetical. Aligned segments represent 93% or greater BLAST matches

In the assemblies, 5 were predicted to contain this GEI, each having some variation in the annotation of the region as seen in Fig. 6. Among the short reads, when evaluated in the limited region of diversity near the 5’ end (as described in the methods), 131 isolates were predicted to carry GEI4. This GEI consistently appeared in the genomes between positions 1.45 Mb and 1.51 Mb, and was flanked by 5-formyltetrahydrofolate cyclo-ligase (sometimes with a hypothetical protein) at the 5’ end and tripartite tricarboxylate transporter permease at the 3’end. It was also widely dispersed in the phylogenetic tree, occurring in nearly every cluster of isolates throughout as seen in Fig. 3.

Among TA isolates there were 32%-53% coverage across the entire GEI and all isolates had 92% or greater identity.

#### Genomic island 5

**Fig. 7:**
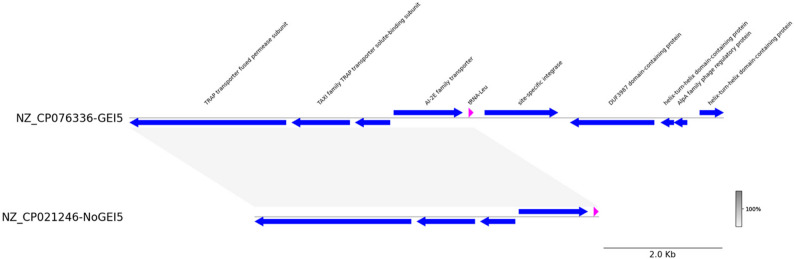
Alignment of GEI5 with sequence commonly located in the same region. The top sequence was predicted to be a genomic island by IslandCompare and IslandViewer4, while the bottom sequence was not and is representative of most TE sequences. Genes without annotation are hypothetical. Aligned segments represent 80% or greater BLAST matches

GEI5 was predicted in only 2 assemblies by IslandCompare and was also the least common GEI in the short reads, with only 11 isolates having more than 99% coverage while the remainder of the short-read sequences had less than 61% coverage. Alignment of the annotated region between the two coverage groups is visible in Fig. 7. Like the other GEIs, this was consistently located in the genomes beginning near 0.91 Mb and flanked in the 5’ region by *lexA* and intergenic region followed by a hypothetical protein in the 3’ region. The dispersion in the TE tree can be seen in Fig. 2 and is expectedly limited.

TA isolates had 24% − 49% coverage of this region with 88% or greater identity.

#### Potential genomic island 6

**Fig. 8:**
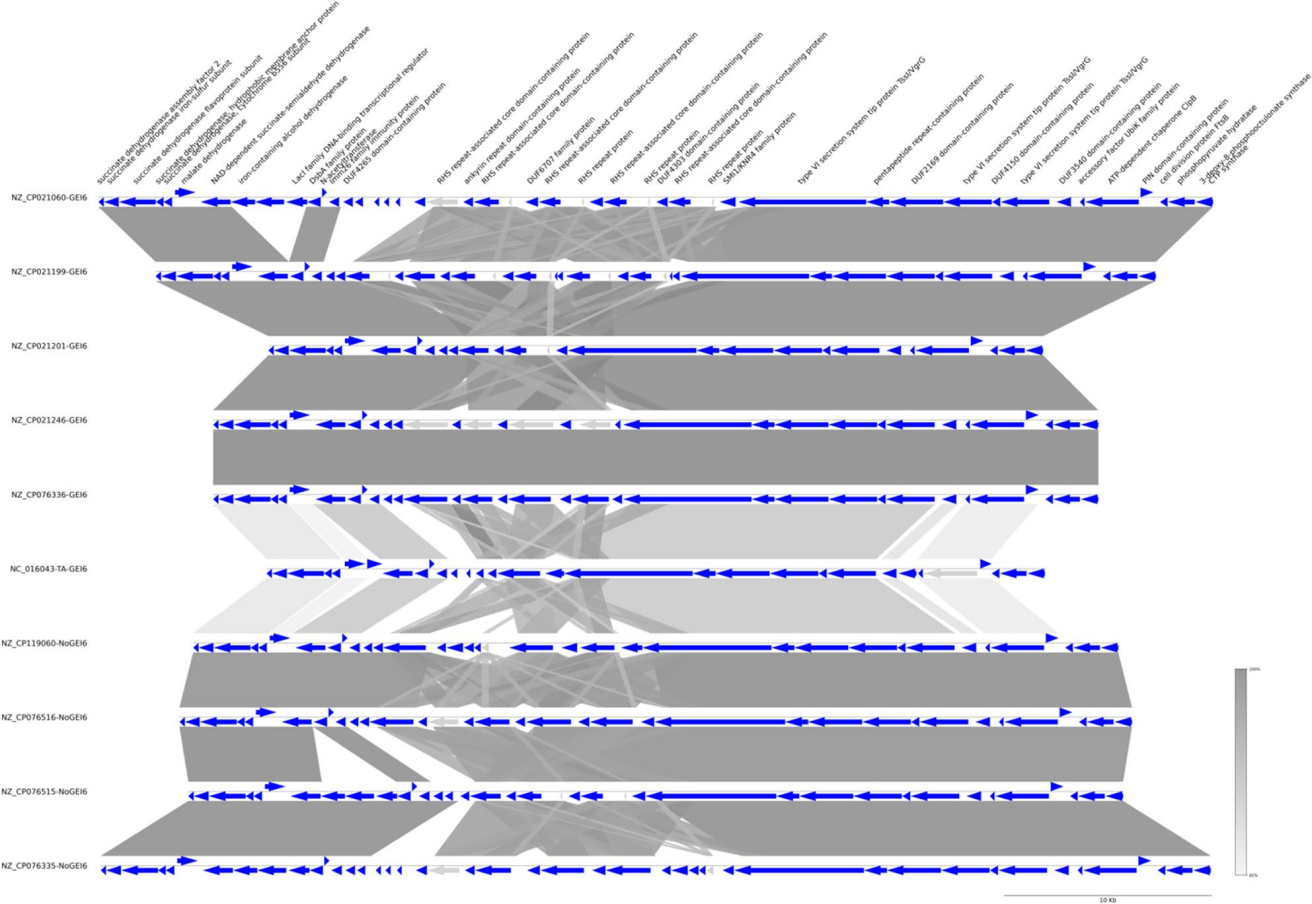
Alignment of Potential GEI6. Included are all 5 genomes where GEI6 was predicted, the homologous region from TA isolate MCE3, and homologous regions from several assemblies where the GEI was not predicted. Genes without annotation are hypothetical. Aligned segments represent 81% or greater BLAST matches

IslandViewer4 identified a potential sixth GEI in 5 of the 12 assemblies. Among the 5 assemblies, a MAFFT alignment indicated 61% identity. Despite the lower percent identity, the annotations between these 5 isolates in the identified region were highly similar. However, they were also highly similar to isolates where the GEI was not predicted. Extraction of homologous regions from all the assemblies revealed similar annotations across all isolates with nucleotide variation limited to discrete segments. No specific variation or pattern was linked solely to isolates predicted to have GEI6, preventing differentiation between predicted presence and absence. Additionally, all short-read TE isolates had 86% or more coverage in this region, while TA isolates had 50.3–78.0% coverage of this same region. This represents the only prediction in the TE isolates to also have significant coverage in the TA genomes. Annotations of the region are outlined in Supplemental Table 1, with observed variation depicted in Fig. 8. The list of annotations from all variations is available in Supplemental Table 5. These annotations included TssI/VgrG proteins, immunity proteins, RHS domains, ankyrin repeat domain-containing protein, and various proteins with enzyme domains. Further analysis identified that the NAD-dependent succinate-semialdehyde dehydrogenase is a PAAR domain-containing protein.

#### *T. asinigenitalis* genomic island

The GEI in the TA isolate was distinct from the TE GEIs. It was the largest GEI identified in this study, with 69 annotated genes across 53,195 bp (Table 2). Of these, 41 encoded hypothetical proteins, making it difficult to elucidate the function of this GEI. Of the genes that were annotated, 3 genes appear in duplicate including *ninB*, *ydaS*, and a gene encoding an S24 family peptidase.

**Table 2:** Annotations of the TA GEI. Multiples of hypothetical proteins are listed for appearance purposes

	TA - GEI Annotations
	GNAT family *N*-acetyltransferase CDS
	NAD(P)H-quinone oxidoreductase CDS
	tpiA CDS
	secG CDS
	tRNA-Leu
	site-specific integrase CDS
2 x	hypothetical protein CDS
	DNA adenine methylase CDS
	helix-turn-helix domain-containing protein CDS
	ssb CDS
	hypothetical protein CDS
	phage protein CDS
	hypothetical protein CDS
	siphovirus Gp157 family protein CDS
	ATP-binding protein CDS
3 x	hypothetical protein CDS
	S24 family peptidase CDS
	YdaS family helix-turn-helix protein CDS
	recombination protein NinB CDS
	hypothetical protein CDS
	ParB N-terminal domain-containing protein CDS
	DEAD/DEAH box helicase family protein CDS
	toprim domain-containing protein CDS
	DUF1064 domain-containing protein CDS
7 x	hypothetical protein CDS
	terminase small subunit CDS
	terminase family protein CDS
12 x	hypothetical protein CDS
	DUF6682 family protein CDS
7 x	hypothetical protein CDS
	glycoside hydrolase family 104 protein CDS
	LPD38 domain-containing protein CDS
	hypothetical protein CDS
	lysozyme CDS
	hypothetical protein CDS
	DUF1902 domain-containing protein CDS
	S24 family peptidase CDS
	YdaS family helix-turn-helix protein CDS
	recombination protein NinB CDS
5 x	hypothetical protein CDS

The coverage of this GEI varied in the TA short-read isolates, 2 samples had 92% coverage, 3 had approximately 41% coverage, and the remainder of the isolates had ≤ 26% coverage of the GEI. TE coverage was low for all samples in this study, with a maximum of 16% and 222 isolates having ≤ 2% coverage.

**Fig. 9:**
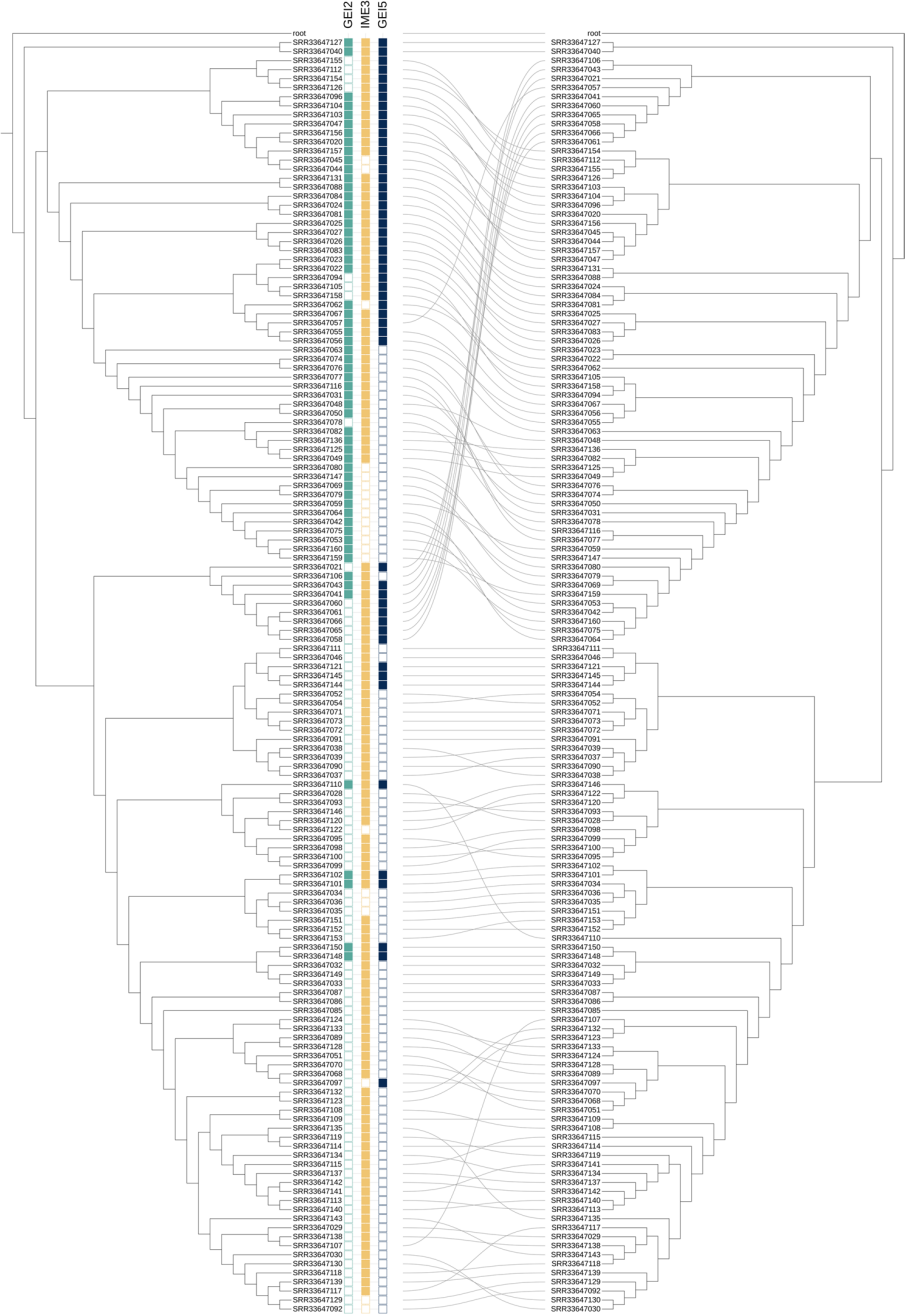
SNP-based Phylogenetic Trees of the Outbreak Samples. The tree on the left is based on high-quality SNPs outside the GEIs only, while the tree on the right is based all high-quality SNPs in all of the genomes. Presence of GEI2, IME3, and GEI5 is indicated for each sample, and the tanglegram indicates position of each isolate within both trees. Branch lengths have been ignored in the tree display to make branching of the tree more visible

### Stability of genomic islands

To illustrate the dispersion and varying patterns of GEIs throughout the outbreak tree, and their influence on phylogenetic structure, SNP-based phylogenetic trees are shown in Fig. 9. A phylogenetic tree with the SNPs in the GEIs included can be seen on the right, while the tree on the left has SNPs in the GEIs filtered to remove the influence of these mobile genetic elements on the tree structure. The tanglegram indicates position of each isolate within both trees. Using the cutoffs established above, 100% of this outbreak dataset contained partial coverage of GEI1, ranging from 57 to 76% coverage, which was consistent with the results seen from isolates in the global dataset described above. Potential GEI6 ranged in coverage from 89 to 90%, also consistent with the results seen in isolates from the global dataset, and was considered present in all isolates. GEI2, IME3, and GEI5 were detected in 42%, 85%, and 36% of the outbreak dataset, respectively, while no isolates contained GEI4. It is of interest to note that 4% of these isolates contained only partial GEI1 and GEI6, and 22% contained all 3 GEIs identified. The remainder of isolates were distributed between different patterns of GEIs with the exception of only GEI5 with partial GEI1 and GEI6, which wasn’t observed in any isolates. Table 3 shows the patterns of GEIs observed.

**Table 3:** GEI Presence and Patterns of GEIs in the Outbreak Dataset. All samples had a partial GEI1 and no samples had a GEI4

Occurrence of GEIs in the Outbreak Isolates								
*Pattern*	Partial GEI1, GEI2, IME3, GI5, GEI6	Partial GEI1, GEI2, IME3, GEI6	Partial GEI1, GEI2, GEI5, GEI6	Partial GEI1, IME3, GEI5, GEI6	Partial GEI1, GEI6 & GEI2 Only	Partial GEI1, GEI6 & IME3 Only	Partial GEI1, GEI6 & GEI5 Only	Partial GEI1 & GEI6 ONLY
*Count*	31	13	3	17	12	60	0	6
*Percentage*	22%	9%	2%	12%	8%	42%	0	4%

## Discussion

TE is of concern in equine trade due to its potential for causing disease, often resulting in acute infertility in mares and economic loss in the breeding industry. The severity of clinical signs can vary widely, ranging from undetectable to severe, even in more recent cases [47]. To better predict strain virulence, it is essential to characterize virulence factors and potential recombination mechanisms. By combining closed genomes with short-read sequencing data from isolates in this study, we constructed a more accurate phylogenetic framework for TE. This comprehensive genomic analysis enables systematic comparisons of clade-specific characteristics and provides critical foundational data for developing improved molecular diagnostics and epidemiological tracking systems for this pathogen.

The GEIs identified in this study mark the first identification of mobile genetic elements in *Taylorella* spp. and represent the potential for recombination between genomes. As in other organisms, these GEIs demonstrate conserved sequences across many branches of the phylogenetic tree occur at discrete locations within the genomes and possess the genetic mechanisms for mobility; however, this genus contains many hypothetical proteins. Functional understanding of these genes may elucidate additional roles of these GEIs. The genes that are annotated (or contain a functional domain) represent potential functions in virulence and ecological fitness.

GEI2 contains *virB3* and *virB8-11*, often associated with transcription of virulence factors as well as being components of T4SSs [13, 48]. Additional T4SS proteins without further classification were also present. Among the annotated genes and functional domains beyond the T4SS were an AbiEii/AbiGii toxin family protein as well as a type III toxin-antitoxin ToxN/AbiQ family protein, also seen in GEI1. Noteably, plasmid mobilization protein mobC was also present along with 2 conjugal transfer proteins, site-specific integrase, RepA, and ArdC and AlpA family proteins.

Many of the proteins annotated in GEI2 are known to be involved in mobilization and conjugation, while others are anti-defense mechanisms as mentioned previously [49, 50]. The AlpA phage regulatory protein has been associated with regulation of cell lysis [51]. T4SSs have been associated with virulence and organism ecology [22].

IME3 and GEI4 are less unique. Like GEI2, IME3 contains *mobC* and AlpA (also found in GEI5), making its contents duplicative of other GEIs. Although, these genes duplications appear to be homologs and not exact duplication events. The GEI4 region primarily appears to consist of genes involved in metabolism and possibly catabolism with the clear exemption of TssI/VgrG, a T6SS spike protein and the associated PARR domain (like GEI6) [52–54].

GEI5 annotations in Supplemental Table 4 include genes spanning a range of functions. In addition to the AlpA discussed previously, also present is a TRAP transporter, annotated as a fused permease subunit and a solute-binding subunit, is commonly associated with nutrient uptake from the environment; while the AI-2E transporter belongs to a superfamily that has little published data, but has been associated with signaling pathways [55, 56].

GEI4 and the potential GEI6 share structural similarities, particularly in their use of RHS domains and the absence of tRNAs. They also do not deviate from the GC content of the whole genome like other GEIs, as seen in Table 1, other GEIs have approximately 3–7% less GC content from the whole genome average [57]. Notably, the T6SS protein domain TssI, the spike protein, is found in both these GEIs with accompanying PAAR domains. These domains are highly conserved across all isolates, regardless of GEI predictions, and are often virulence determinants in organisms [13]. In the potential GEI6, several immunity protein domains are also conserved, extending across the genus, which may preclude this region from being a genomic island, as they are generally species specific. However, these immunity proteins are associated with polymorphic immunity systems or heterogenous polyimmunity loci. In these systems, the spike protein is a common method for delivering bactericidal effectors into neighboring cells with the help of immunity proteins that prevent the cell’s own death. These systems clearly enhance an organism’s virulence with the ability to deliver toxins and/or effectors into neighboring cells [58–60]. It does, however, remain unclear if GEI6 is a true genomic island with mobility. The ubiquitous nature of many conserved proteins domains in this diverse dataset may be indicative that it has become fixed in the genome, but it is clearly a site with significant diversity, and the source of this diversity could not be determined in this analysis.

Many of the genes within these predicted GEIs are likely to have additional functions, particularly anti-defense, aiding this fastidious organism in surviving and thriving within competitive environments, such as the male reproductive tract. However, the data set includes only a limited number of isolates that have been characterized in terms of clinical signs, virulence levels, and other phenotypic traits. This limitation prevents any definitive correlation between the GEIs identified in this study and phenotypic data. Further research is essential to determine whether these GEIs are linked to environmental stressors or variations, and to explore how they might impact virulence or enhance the organism’s survivability.

The recent U.S. outbreak of CEM, which resulted from a single introduction with clonal expansion, according to molecular epidemiological studies as well as epidemiological tracing, provided an opportunity to examine GEIs identified in this study for potential mobility under natural conditions. This outbreak has occurred primarily among geldings through fomite transmission with numerous opportunities for re-exposure of animals over a period of several years, limiting the conclusions that can be drawn from this data beyond demonstrating the organism’s ability to excise or acquire these GEIs. The various patterns observed may well be correlated with usefulness of genes contained in the GEIs present and their ability to improve survivability of the TE strain.

Further investigation into the functional potential of these genomic islands could enlighten our understanding of TE’s pathogenicity and potentially reveal insights into the pathogenic potential of TA.

## Conclusions

This work demonstrates several genomic islands, their associated genes, and their distribution throughout a more complete phylogenetic tree. This is the first documentation of mobile genetic elements in this genus. This study was limited to genomic island prediction and did not extend to other recombination events. Detection of candidate recombination regions across the dataset was not pursued, as available bioinformatics tools for this purpose require further development.

## Supplementary Information

Supplementary Material 1: Supplemental Fig. 1. IslandViewer4 Genome Maps.

Supplementary Material 2: Supplemental Fig. 2. Electronic format of Taylorella kSNP trees in Fig. 1.

Supplementary Material 3: Supplemental Fig. 3. Electronic format of core TE tree in Fig. 2.

Supplementary Material 4: Supplemental Table 1. Long-read statistics for isolates closed for this project., Supplemental Table 2. NCBI Accession List., Supplemental Table 3. Presence and absence of GEIs in assemblies., Supplemental Table 4. Annotations of GEI1 through 5., Supplemental Table 5. Potential GEI6 annotation list from each variation.

## Data Availability

Data is publicly available from NCBI. A full list of accessions for all assemblies and raw data used in this research is available in Supplemental Tables 1 and Supplemental Table 2.
