# Supplementary material for: Initial identification of genomic islands in Taylorella equigenitalis and Taylorella asinigenitalis and their distribution in isolates from around the world
Source: BMC Microbiol. 2026 Jun 5;26:728. doi: 10.1186/s12866-026-05204-3 (PMC13474744; doi:10.1186/s12866-026-05204-3)
Supplement: Supplementary file 1 [file 12866_2026_5204_MOESM2_ESM.pdf]

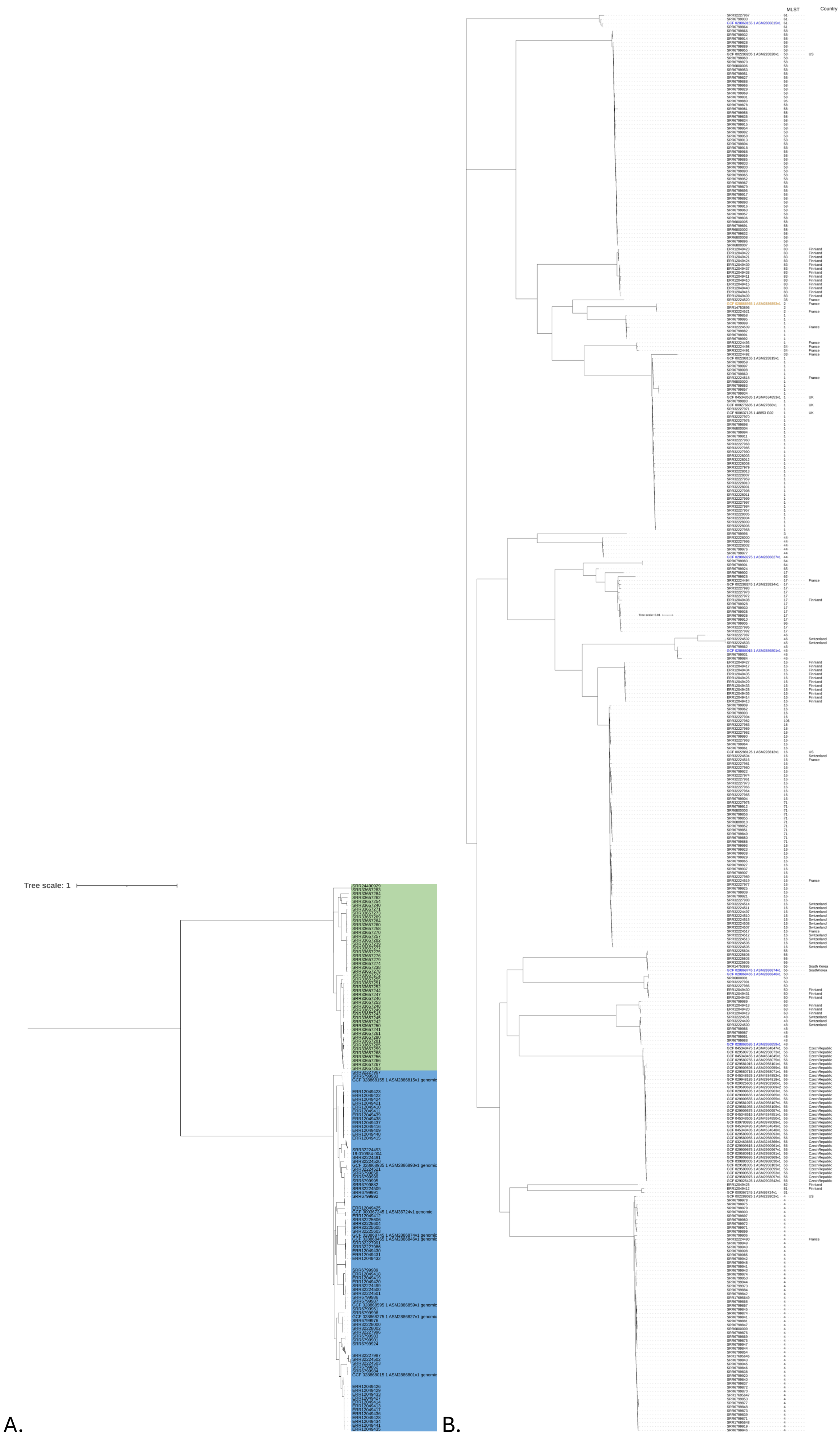
Tree scale: 1
MLST
Country
A.
B.
